# Maternal Near-Miss and Maternal Mortality in a Tertiary Care Center of Western Uttar Pradesh: A Retrospective Study

**DOI:** 10.7759/cureus.42697

**Published:** 2023-07-30

**Authors:** Aruna Verma, Renu Choudhary, Rachna Chaudhary, Monika Kashyap

**Affiliations:** 1 Department of Obstetrics and Gynecology, Lala Lajpat Rai Memorial (LLRM) Medical College, Meerut, IND

**Keywords:** severe maternal outcome ratio, mortality index, maternal near miss incidence ratio, maternal near miss, maternal mortality ratio, maternal mortality

## Abstract

Introduction: Maternal mortality is an important indicator to assess the quality of services provided by the health care system. However, maternal near-misses as well as maternal mortality are also indicators of how well the health care system serves pregnant women. To improve our healthcare system in terms of investigative capacity, infrastructure, and personnel, a near-miss registry can provide important information on gaps in pregnancy facilities. This will help us to identify the requirements for referral facility improvements and the need for various health awareness programs. We, therefore, designed this study to analyze the various near-miss events in mothers and compare them with maternal mortality.

Methods: Present study was conducted in the Department of Obstetrics and Gynecology, Lala Lajpat Rai Memorial (L.L.R.M.) Medical College associated with Sardar Vallabh Bhai Patel (S.V.B.P.) Hospital Meerut, Uttar Pradesh (UP), India for a period of one year and data were collected retrospectively from January 2022 to January 2023. All patients with life-threatening conditions such as excessive bleeding during pregnancy, hypertensive disorders of pregnancy (HDP), and septicemia that occurred during pregnancy or childbirth or within 42 days of termination of pregnancy and required ICU admissions, were included in the study. The total number of deliveries during the study period was 4,360 with 4,333 live births (LB). The total number of eligible cases was 79, out of which 52 were identified as maternal near misses and 27 were maternal mortality. Various maternal mortality and near-miss indices were analysed and statistical analysis was done using the SPSS version 21 (IBM Corp., Armonk, NY, USA).

Results: Our hospital's maternal mortality ratio (MMR) was 623/1lakh (0.623%), which is higher than the probability due to the deficiency of appropriate medical services in the nearby areas of western UP. The number of maternal near misses per 1000 LB (maternal near-miss ratio [MNMR]) was 12/1000 LB and the severe maternal outcome rate (SMOR) was 18/1000 LB (1.82%). In our study, hemorrhage and hypertensive disorder in pregnancy were the leading cause of morbidity and mortality followed by sepsis and severe anemia. Among organ dysfunction cardiac illness followed by respiratory dysfunction was the leading cause of morbidity and mortality.

Conclusion: It is clear that there is a high burden of maternal near-miss in developing countries. There should be the establishment of well-equipped referral units at the periphery with trained manpower. The establishment of obstetrical high-dependence units (HDUs), rapid availability of blood and blood products, training of staff, and availability of multidisciplinary teams can minimize maternal mortality and morbidity.

## Introduction

A maternal near miss (MNM) is a condition in which a woman, regardless of the location or duration of her pregnancy, nearly dies from a pregnancy or delivery problem within 42 days of termination of pregnancy, but survives because of the care she received or because of an accident [1]. Maternal mortality is an important indicator to assess the various services provided by health facilities. The World Health Organization (WHO) first defined surveillance recommendations for monitoring near miss in 2009 and published the WHO near-miss approach for maternal health in 2011 [2,3].

All over the world, there is a gradual decrease in the maternal mortality ratio (MMR), and it decreased by 38% from 342 in the year 2000 to 211 in the year 2017 per 100,000 live births [4]. It is also declining in India and has reached from 130 during 2014-2016 to 122 during 2015-2017, but in Uttar Pradesh, the MMR was 216/100,000 live births in 2015-2017 [5]. In India MMR falls well short of the standards needed to achieve the Sustainable Development Goals (SDGs) that countries work together to achieve. In SDG 3, the MMR has to be reduced up to 70 per 100000 live births. Many Indian states have already achieved it, but the majority are still far from it [6]. Health resources must be strengthened to meet national and international goals. Extensive research on obstetric near misses and maternal mortality has been conducted in recent years. These near-death experiences were women who later died, but mortality occurred as a result of a delay in seeking medical attention or for other causes. As a result, they share several characteristics, especially risk factors.

To improve the healthcare system in terms of facility of testing, equipment, and personnel, near-miss registries provide valuable information on deficiencies in healthcare facilities for pregnant women and help to identify the requirements for improved referral facilities and the need for various health awareness programs. In an attempt to follow the WHO mandate to initiate near-miss reviews in all settings in all countries, India’s national technical group in 2014 proposed a set of local-specific expert consensus criteria [7]. We can easily analyze high-risk pregnant women as well as plan and carry out important interventions for obstetrical catastrophes and create awareness programs for good results by effectively implementing the guidelines and the WHO's near-miss concept. As our center is a tertiary care center in western Uttar Pradesh and receives a large number of referral cases from most rural areas and marginal areas of other states, we designed this study at our institute to calculate the frequency of maternal near misses and to compare the types of near misses and maternal mortality.

## Materials and methods

Place and type of study

This was an observational retrospective analytical study conducted in the Department of Obstetrics and Gynecology, Lala Lajpat Rai Memorial (L.L.R.M.) Medical College associated with Sardar Vallabh Bhai Patel (S.V.B.P.) Hospital Meerut, Uttar Pradesh, India for a period of one year and data were collected retrospectively from January 2022 to January 2023. Approval from the Institutional Ethics Committee was taken before collecting data.

Study population and sample size

All patients with life-threatening conditions such as severe postpartum hemorrhage, severe pre-eclampsia, eclampsia, sepsis or severe systemic infection, rupture uterus, ruptured ectopic pregnancy that occurred during pregnancy or childbirth or within 42 hours of delivery and underwent critical intervention or needed ICU admissions were included in the study. Their socio-demographic features, mode of delivery, diagnosis on admission, surgical intervention, intervention, ICU admissions, duration of hospital stay, and outcome were collected from the record available in the hospital’s record section. The patient’s characteristics including parity, age, and gestational age on admissions were also recorded. Apart from the conditions of the patient during admission, the primary cause of morbidity, mode of delivery, the consequences of morbidity, organ failure or dysfunction, and the key interventions taken to save her, and follow-up during hospital stay were also noted down.

Near-miss events were recorded based on the Health and Family Welfare Government of India Guideline (2014). Patients were categorized by final diagnosis with respect to hemorrhage, hypertension, sepsis, anemia, thrombocytopenia, and other medical disorders that were considered as indirect causes contributing to MNM and death.

All women who met the criteria for maternal near-miss cases or those who died (i.e., women presenting a severe maternal outcome) were referred to as women with life-threatening conditions (WLTC). It is the total of maternal near-misses and maternal deaths (MD) (WLTC=MNM+MD).

Severe maternal outcome ratio (SMOR) referred to the number of women with life-threatening conditions (MNM+MD) per 1000 live births (LB) (SMOR=[MNM+MD]/LB). MNM ratio (MNMR) referred to the number of maternal near-miss cases per 1000 live births (MNMR=MNM/LB). Maternal near-miss mortality ratio (MNM:MD) referred to the ratio of MNM cases to MD. Mortality index (MI) referred to the number of maternal deaths divided by the number of women with life-threatening conditions expressed as a percentage (MI=MD/[MNM+MD]).

The ratio of maternal near misses to maternal mortality and the mortality index provides information about the health care at a particular facility. A higher maternal near-miss mortality ratio indicates better hospital services and care.

Statistical analysis was done by using SPSS version 21 (IBM Corp., Armonk, NY, USA).

## Results

During the study period, there were 4,360 deliveries at our institute with live births of 4,333. The total number of eligible cases was 79 out of which 52 were identified as maternal near-miss cases and 27 were maternal mortality registered during the study period (Table 1).

**Table 1:** Maternal near-miss and mortality indices

Indices	Numbers
Total number of deliveries	4360
Total no. of live births (LB)	4333
Number of near-miss cases (MNM)	52
Number of maternal mortality cases (MM)	27
Maternal near miss incidence ratio (MNM IR=MNM/LB)	12/1000LB
Maternal mortality ratio (MMR=MM/LB)	623/1lakh LB (.623%)
Maternal near miss: maternal mortality ratio (MNM:MD)	1.9:1
Mortality index (MD/MNM+MD)	34.1%
Severe maternal outcome ratio (SMOR=MNM+MD/LB)	18/1000 LB (1.82%)

In demographic parameters, the maximum number of near-miss cases belonged to the Muslim religion (63.46%) compared to the Hindu religion, and the maximum mortality was seen in the Hindu (55.56%) religion. Approximately 96 % of the near miss and 100% of the mortality group were unbooked and 94% of near-miss and 62% of maternal mortality belonged to lower socioeconomic groups. In the study 78.84% were near-miss and all cases of maternal mortality were illiterate (Table 2).

**Table 2:** Socio-demographic characteristics of women SMO- Severe Maternal Outcome MNM- Maternal Near-Miss MM- Maternal Mortality

	SMO(n=79)	MNM(n=52)	MM(n=27)	P-VALUE	MORTALITY INDEX
RELIGION					
MUSLIM	48	33(63.46)	12(44.44)	0.1054	25
HINDU	31	19(36.54)	15(55.56)		48.3
2 . BOOKING STATUS					
BOOKED	2	2(3.84)	0	0.3037	0
UNBOOKED	77	50(96.15)	27(100)		35.0
3. SOCIO-ECONOMIC STATUS (MODIFIED KUPPUSWAMY SCALE)					
LOWER MIDDLE	13	3(5.70)	10(37.03)	0.0002	76.9
UPPER MIDDLE	3	1	2		0
LOWER	63	48(94.23)	15(62.96)		25.7
4. EDUCATIONAL STATUS					
LITERATE	11	11(21.15)	0	0.009	0
IILITERATE	68	41(78.84)	27(100)		39.7

Both morbidity (28%) and mortality (37%) were maximum in the age group of 20-24 years and maternal near-miss was higher (76.92%) in multigravida patients. Maternal near-miss (53.84), as well as maternal mortality (51.85), were maximum between 32-34 weeks of gestation. Higher morbidity was seen in the cases with one previous lower segment cesarean section (LSCS) (13%) and mortality was higher in both previous one and previous two LSCS cases (7%) (Table 3).

**Table 3:** Selected reproductive characteristics of the subjects SMO- Severe Maternal Outcome MNM- Maternal Near-Miss MM- Maternal Mortality ANC- Antenatal Case PNC- Postnatal Case LSCS- Lower Segment Cesarean Section

1. AGE (YEARS)	S.M.O (N=79)	MNM (N=52)	MM (N=27)	P-VALUE	MOTALITY INDEX
<20YRS	10	8(15.3)	2(7.41)	0.3153	20.0
20-24	25	15(28.85)	10(37.04)	0.4153	40.0
25-29	21	12(23.08)	9(33.33)	0.3421	42.8
30-34	13	8(15.3)	5(18.52)	0.7319	38.4
>35YRS	10	8(15.38)	2(7.4)	0.0764	20
PARITY					
PRIMIPARA	32	20(38.46)	12(44.44)	0.0210	15.1
MUTLIPARA	47	40(76.92)	7(25.92)		8.8
PERIOD OF GESTATION (WEEKS)					
<32	21	14(26.92)	7(25.92)	0.2011	8.8
32-34	42	28(53.84)	14(51.85)	0.0019	17.72
>34	16	12(23.08)	4(14.81)	0.3452	25.0
ANTENATAL/POSTNATAL					
ANC	14	6(11.54)	8(29.63)	0.0064	57.1
PNC	13	12(23.08)	1(3.70)		7.6
LSCS DONE AT ANOTHER FACILITY					
	2	0	2(7..41)	0.0482	100
PREVIOUS PREGNANCY					
PREVIOUS 1LSCS	9	7(13.46)	2(7.41)	0.4220	22.22
PREVIOUS 2LSCS	7	5(9.62)	2(7.41)	0.7432	28.57
PREVIOUS 3LSCS	1	0	1(3.70)	0.1660	100

Obstetrical hemorrhage was the leading cause of near-miss cases (76.92%) either because of early pregnancy hemorrhage (abortion and ectopic pregnancy) or antepartum/postpartum (APH/PPH) hemorrhage. Whereas in the mortality group, hypertensive disorders of pregnancy (HDP) were the leading cause (Table 4).

**Table 4:** Primary disorders causing maternal mortality and maternal near-miss SMO- Severe Maternal Outcome MNM- Maternal Near-Miss MM- Maternal Mortality APH- Antepartum Hemorrhage PPH- Postpartum Hemorrhage HDP- Hypertensive Disorders of Pregnancy

PRIMARY CAUSE	SMO( n=79)	MNM(n=52)	MM(n=27)	MI
1. HEMORRHAGE	47	40(76.92)	7(25.9)	14.8
EARLY PREGNANCY	9	9	0	0
ABORTION		3(3.75)	0(0.00)	
ECTOPIC PREGNANCY		6(11.54)	0(0.00)	
2. APH	21	16	5	23.8
PLACENTA PREVIA		11(21.15)	2(7.41)	
ABRUPTION		5(9.6)	3(11.11)	
3. PPH	17	15	2	11.7
PLACENTA ACCRETA SPECTRUM		6(11.53)	0(0.00)	
RUPTURE UTERUS		9(17.30)	2(7.41)	
4. HDP	32	20(38.46)	12(44)	37.5
ECLAMPSIA		9(17.30)	5(18.52)	
PREECLAMPSIA		11(21.15)	7(25.93)	

In cases with organ dysfunction/life-threatening conditions, we found that both in near-miss cases and mortality group patients admitted with one or more organ dysfunction and life-threatening conditions on admissions. Here septicemia was the commonest (59.61%) in the near-miss group and cardiovascular causes and septicemia were the commonest in the mortality group (Table 5).

**Table 5:** Organ dysfunction/life-threatening conditions SMO- Severe Maternal Outcome MNM- Maternal Near-Miss MM- Maternal Mortality

	SMO (N=79)	MNM(n=52)	MM(n=27)
SEVERE ANEMIA	38	27(51.92)	11(40.74)
SEPTICAEMIA	48	31(59.61)	17(62.96)
CARDIOVASCULAR (SHOCK, CARDIAC ARREST)	30	13(25)	17(62.96)
RESPIRATORY DYSFUNCTION	15	8(15.38)	7(25.93)
RENAL CAUSES	11	4(7.69)	7(25.93)
HAEMATOLOGICAL DYSFUNCTION	23	10(15.38)	13(48.14)
CENTRAL NERVOUS SYSTEM	18	9(17.31)	9(33.33)

In pregnancy outcomes, cesarean section accounted for 34.6% and 37% in the near-miss and mortality group as compared to 3.8% and 11% in vaginal delivery. The clinical lifesaving intervention was required in both groups. ICU admissions and use of cardio tonics/vasopressors were needed in 96% of near-miss cases, exploratory laparotomy was done in 15% of near-miss groups. In the maternal mortality group, 92% required ICU admission, 85% required vasopressors, and 77% needed intubation/resuscitation measures one case required hysterectomy and internal iliac ligation (Table 6).

**Table 6:** Pregnancy outcome and clinical life-saving intervention MNM- Maternal Near-Miss MM- Maternal Mortality LSCS- Lower Segment Cesarean Section ICU- Intensive Care Unit

PREGNANCY OUTCOME	MNM	MM
LSCS	18(34.6)	10(37)
VAGINAL DELIVERY	2(3.85)	3(11.1)
CLINICAL LIFE-SAVING INTERVENTION		
MANUAL REMOVAL OF PLACENTA	3(5.77)	0.00
HYSTERECTOMY	7(13.46)	1(3.70)
INTERNAL ILIAC ARTERY LIGATION	5(9.6)	1(3.70)
EXPLORATORY LAPAROTOMY	8(15.38)	0
REPAIR OF GENITAL INJURIES	3(5.76)	0
RESUSCITATION PROCEDURE/INTUBATION	49(94.2)	21(77.77)
USE OF CARDIOTONICS/VASOPRESSOR	50(96.1)	23(85.18)
ICU ADMISSIONS	50(96.1)	25(92.5)
BLOOD TRANSFUSION	48(92.3)	25(92.5)

## Discussion

The MMR at our hospital was 623/1lakh (0.623%) during the study period, which is higher than the probability due to bad health infrastructure and other services in the nearby areas of western UP, especially in the remote rural areas. In our study, the MNMR was 12/1000 LB, with 18/1000 LB (1.82%) of severe maternal outcome ratio (SMOR). We have reviewed many studies from different parts of India and noticed that they used different criteria for comparison.

India is a resource-limited county, where women suffer more than in European countries especially due to shortcomings in managing obstetrics catastrophes at various levels. There are three delays, which are considered one of the main reasons for bad obstetrics emergency care in India. The first is lack of awareness which leads to delays in availing health care services. The next one is due to a lack of accessibility to health care facilities, transport, cost, and sometimes socioeconomic problems. The third delay is a lack of appropriate care at the health care center, delay in diagnosis or decision making, or deficiency of resources or trained health caregivers. In the present study, we found that 27 cases of maternal mortality and 52 cases of near misses had more than one or more organ dysfunction. We found that first and second delays were the most common reason for high mortality and morbidity. A review done by Visi and Akoijam showed that most of the near-miss were due to delays in the decision to seek health care. Lack of knowledge of the warning signs of pregnancy plays an important role in the delay of management [8]. The majority of them were unbooked and belonged to lower socio-economic backgrounds as well as illiterate. Sarma and Kalita showed that one woman died for every four women who survived with life-threatening complications. They also proved that early evaluation and then early referral from the primary health centers is the most important step to save the mother as well as the baby [9].

In our study, hemorrhage and hypertensive disorder in pregnancy were the leading cause of morbidity and mortality followed by sepsis and severe anemia. Rulisa et al. showed that the most common cause of MNM was peritonitis, hypertensive disorder in pregnancy, and hemorrhagic diseases [10]. Antepartum hemorrhage and pre-eclampsia with severe features constituted the major complications among the hemorrhagic disease group and in the hypertensive disease group. Among organ dysfunction cardiac illness followed by respiratory dysfunction was the leading cause of morbidity and mortality.

Over the last decade, MNM gaining momentum as an indicator of obstetric care. Different criteria have been used in the past to define MNM, like management-specific criteria, disease-specific criteria, or organ failure/dysfunction criteria. WHO criteria defined near miss by using organ dysfunction criteria as a standard in identifying near miss cases. Many studies have been done to audit the MNM cases in India and worldwide. Presently, the focus has shifted to maternal near misses from maternal mortality as an important indicator of maternal wellbeing.

A list of various studies from different parts of India on maternal near misses is shown in Table 7.

**Table 7:** Various studies on maternal near-miss from different parts of India

Author	Setting	Criteria for near miss	Live birth	Maternal mortality ratio	Maternal near miss mortality ratio	Mortality index	Near miss conditions	Mortality’s cause
Roopa PS et al. 2013 [11]	Tertiary care hospital, Manipal	Disease-specific management based	7330	313	17.8	14.9	Hemorrhage(44.2%)	Sepsis(52%)
Bakshi et al. 2015 [12]	Mutlicentric trial, Dehradun	Disease-specific	688	1453	74	16.4	Sepsis(58.8)	Hemorrhage(37.2%)
Rathod et al. 2016 [13]	Tertiary care center, Yavatmal	Organ dysfunction	22092	298.7	7.3	29.07	Hemorrhage(26.7)	Severe anemia
Tallapureddy et al. 2017 [14]	Medical college, Hyderabad	Disease-specific management based	3784	158.5	8.4	15.79	Hemorrhage(40.5)	Hypertension(48%)
Mansuri et al. 2019 [15]	Medical college, Indore	Organ dysfunction	21491	367.6	11.49	24.23	hypertension	Hypertension hemorrage
Verma et al. 2020 [16]	Tertiary care hospital, Etowah	Disease-specific	8638	891.4	16.6	34.8	Hypertension(45.7)	Sepsis(54%)
Our study 2023	Tertiary care center, Meerut	Disease-specific	4333	623	1.9:1	34.1	Hemorrhagic disease	Hemorrhage Septicaemia

## Conclusions

This study clearly showed the high burden of near-miss mortality in developing countries. Any long- and short-term health planning should bear in mind to tackle the challenges in developing countries. There are few health centers in rural areas that only offer poor services and lack equipment. Government should strengthen the primary health centers with better equipment and train all healthcare workers so that sick patients can be resuscitated and referred after stabilization to reduce morbidity and mortality. There should be the establishment of well-equipped referral units at the periphery with trained manpower. The establishment of an obstetrics high dependency unit (HDU), availability of blood and blood products from the blood bank, training of all the staff, and availability of a multidisciplinary team can reduce maternal morbidity and mortality. National standardized definitions, Standard Operating Procedures (SOPs), and guidelines for referral should also be made to improve maternal outcomes.
